# Coupled Folding and Specific Binding: Fishing for Amphiphilicity

**DOI:** 10.3390/ijms12031431

**Published:** 2011-02-24

**Authors:** Vikas P. Jain, Raymond S. Tu

**Affiliations:** Department of Chemical Engineering, The City College of City University of New York, 140th Street and Convent Avenue, Steinman Hall T313, New York, NY 10031, USA; E-Mail: vjain@gc.cuny.edu

**Keywords:** peptide design, amphiphilic peptide, dynamic folding, binding kinetics

## Abstract

Proteins are uniquely capable of identifying targets with unparalleled selectivity, but, in addition to the precision of the binding phenomenon, nature has the ability to find its targets exceptionally quickly. Transcription factors for instance can bind to a specific sequence of nucleic acids from a soup of similar, but not identical DNA strands, on a timescale of seconds. This is only possible with the enhanced kinetics provided for by a natively disordered structure, where protein folding and binding are cooperative processes. The secondary structures of many proteins are disordered under physiological conditions. Subsequently, the disordered structures fold into ordered structures only when they bind to their specific targets. Induced folding of the protein has two key biological advantages. First, flexible unstructured domains can result in an intrinsic plasticity that allows them to accommodate targets of various size and shape. And, second, the dynamics of this folding process can result in enhanced binding kinetics. Several groups have hypothesized the acceleration of binding kinetics is due to induced folding where a “fly-casting” effect has been shown to break the diffusion-limited rate of binding. This review describes experimental results in rationally designed peptide systems where the folding is coupled to amphiphilicity and biomolecular activity.

## 1. Introduction

Configurational dynamics in proteins have been found to be critical to a variety of physiological processes [1,2] such as transcription and translation regulation [3], cellular signal reduction [4], protein phosphorylation [5] and molecular assemblies [6]. The cooperative processes of folding and binding are important to many of these biological processes and include several intra- and inter-molecular factors that determine the interaction dynamics between the proteins and their targets [7]. Due to complexities involved in these interactions and protein folding, engineering simple hybrid peptide designs has emerged as an attractive tool to understand protein/peptide structure, function and dynamics. This review describes the use of amphiphilic α-helices, a commonly-observed structural feature found in many proteins and biologically active peptides. This structural motif has been found to play multiple roles in protein folding, protein-protein recognition, protein-membrane interactions, and protein and peptide biological activity [8]. The amphiphilic architecture is also important for the stabilization of the secondary structure of peptides/proteins when they bind to apolar surfaces such as phospholipids membranes, air *etc* [9,10]. While a plethora of studies have examined the structural role of these helices in proteins, there remains a paucity of studies that examine the critical role of dynamics in the functional role of amphiphilic α-helices.

Designing biomimetic peptides from natural analogues requires a lucid perspective on naturally occurring protein folding and binding as cooperative processes [2,11–13], where the protein searches for favorable intramolecular or intermolecular interactions [14–16]. There are many proteins in nature that are disordered in their physiological condition, but when they bind to the specific target or site, they become more ordered [11,17–19]. This phenomenon of coupled folding to binding has been shown to enhance the binding kinetics and the selectivity of the overall binding process [16,20]. Induced folding of the protein results in key structural and thermodynamic changes. First, flexible unstructured domains with an intrinsic plasticity allow proteins to accommodate targets of various size and shape; and second, free energy of binding is required for compensation for the entropic cost of ordering of the unstructured region. A site that does not provide enough binding free energy cannot induce folding and, hence, cannot form stable complex.

**Figure 1.**
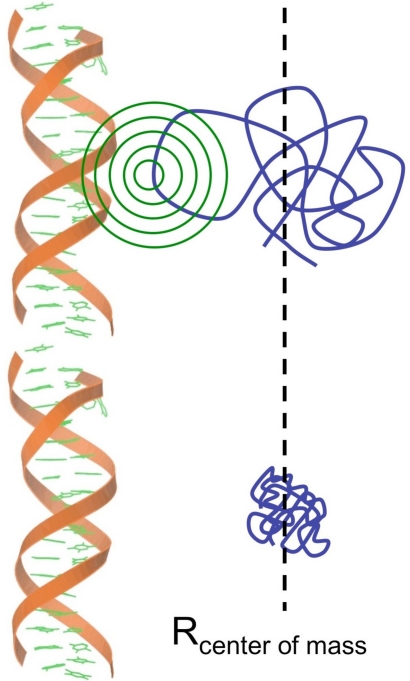
A cartoon representing the increase in the kinetics of the binding process due to fly-casting mechanism (adapted from Shoemaker *et al*. [21]).

While the notion of induced folding provides a thermodynamic framework for the configurational changes associated with the binding of intrinsically disordered proteins, other efforts have examined the influence of kinetics on natively unfolded proteins. Shoemaker *et al*. showed that relatively disordered proteins have a bigger capture radius as compared with the ordered protein molecule and the mobility of the folded protein is restricted due to the fixed conformation, which is not the case with the unfolded protein. In this scenario, an unfolded protein binds weakly to its target site at a relatively large distance followed by the folding of the protein as it approaches the specific binding site. This mechanism results in accelerated binding kinetics and has been coined as the “fly-casting mechanism” (Figure 1) [21,22].

Engineering synthetic peptides with controllable molecular architectures that mimic the natural phenomena observed in this class of proteins requires careful consideration of the dominant inter- and intramolecular interactions. In the next section, we will examine several protein systems and biologically inspired peptide systems that have the ability to bind DNA and RNA, changing states from intrinsically disordered to ordered on binding. In the third section, we will discuss how new designs can be developed based on a set of simple design “rules” for defining the thermodynamics of a synthetic helical peptides. In the fourth section, we will discuss the ability to use these rules in combination with native binding sequences to engineer new amphiphilic peptide analogues.

## 2. DNA/RNA Binding Intrinsically Disordered Proteins

### 2.1. DNA Binding Proteins

A handful of researchers have attempted to use these intrinsically disordered proteins as the models for their peptide design, where peptides are constructed to mimic the dynamics of the binding process observed in nature. These authors are motivated to design peptides that explore the fundamental relationship between the dynamics of the folding and the kinetics of the selective binding that will lead to better understanding of the overall process. Such peptides with accelerated kinetics can be used for designing new a generation of molecules that can be used as rapid acting diagnostic tools. For example, the basic leucine zipper family (bZIP), one of the best characterized family of DNA binding motif, consist of a *N*-terminal basic region that binds to a specific sequence of DNA and *C*-terminal leucine zipper that is responsible for dimerization [23–25]. In the absence of DNA, leucine zipper is helical and dimeric while the basic region is flexible and partially disordered. In presence of sequence specific DNA, the basic region is fully helical, which shows that the folding of the protein is coupled with the binding of its target DNA. The activity of the transcription factor captures both aspects discussed in the prior section; the existence of the intrinsically disordered state yields enhanced kinetics (fly-fishing), and conformational selection is exhibited on target binding.

O’Neil *et al*. designed different peptides based on leucine zipper motif that helps in understanding the interaction of DNA with this class of proteins [26]. An induced helical fork model has been devised suggesting that the role of the leucine zipper is to position the basic region so that the zipper can both interact with DNA and promote the helix formation in the basic regions, where helicity is induced in basic region only in presence of the target DNA. The helical fork model also indentifies the residues that are important for helix stability but do not help in DNA recognition. O’Neil *et al*. replaced these residues with the small, neutral, helix favoring amino acids, showed that the basic region forms the stable helix that binds to the major groove of DNA. The authors also showed that the specificity is maintained as the binding with non-specific DNA does not show rise in helicity of basic region as compared to that with specific DNA. Talanian *et al*. showed that the basic region of the yeast transcriptional activator GCN4, which belongs to the bZIP family, retains the sequence specificity even when the leucine zipper is removed [27]. A peptide was synthesized with the basic region corresponding to the residues 222–252 of GCN4 and the leucine zipper was replaced with the Gly-Gly-Cys liner at the *C*-terminal. This peptide was then dimerized to give a disulphide-bonded dimer and, using DNase I footprinting, they showed that it retains the sequence specificity by comparing the results with the basic region attached to leucine zipper. The minimum length for the basic region sequence for specific binding was also determined [27,28]. Peptides of different sizes of basic region were synthesized with the GGC linker attached to give disulphide-bonded dimer after dimerization. DNase I experiments were conducted, and it was found that the peptides containing only 20 residues of GCN4 basic region show the same sequence specificity as that of intact protein. Also, circular dichroism experiments showed that 15 residues from the basic region of GCN4 (231–245) form a helix when contacting the specific DNA target. This sequence provides a template for peptide designs to study the interactions of a class of DNA-binding peptides where folding and binding are cooperative.

Other authors have taken advantage of this inherent modularity of DNA-binding sequences, combining dimerization domains with DNA binding domains. Kim *et al*. designed a peptide consisting of alternate lysines (KGKGKGK) and the leucine zipper as a dimerization domain. This peptide sequence has been shown to bind specifically to Z-DNA [29]. Tu *et al*. modified the bZIP sequence by adding alkyl tails to the C-terminus that formed mono- and dialkyl bZIP peptide-amphiphiles and investigated the effects of this modification on secondary structure and self-assembly formation [30]. Tu *et al*. observed that the peptide amphiphiles combines the characteristic of the basic zippers and cationic amphiphiles, capturing the functional behavior of both. The peptide amphiphiles enhance secondary structure and form hierarchical structures as they bind to DNA in helical conformation. Peptide amphiphiles, in general, are capable of forming hierarchical assemblies, making them an interesting choice to use as functional building blocks for different systems such as gene delivery and artificial transcriptional factors [31,32].

### 2.2. RNA Binding Proteins

RNA-protein interactions have also gathered attention [33,34]. During this interaction, folding can be induced in RNA alone, protein alone or in both RNA and protein [35–39]. For example, folding is induced in RNA when ribosomal S15 protein binds to rRNA [35], while binding of antitermination protein N of bacteriophage λ with its cognate RNA induces folding in the completely disordered protein [39]. Binding of neuleolin protein with its cognate stem loop RNA induces folding of RNA hairpin loop and the ordering of the two RNA binding domains of the protein [37,38]. Here, flexibility in both RNA and protein is essential for tight binding and also for RNA sequence recognition. In another example, the Tobacco Mosaic Virus coat protein has a 25-residue loop that is natively disordered but undergoes a disorder-to-order transition upon RNA binding during the assembly of the capsid [40–43].

Several authors have shown that Rev protein, an essential regulatory protein encoded by human immunodeficiency virus, has arginine-rich binding regions that are found in many viruses [44–46]. In this case, specific binding of Rev protein to RNA not only stabilizes the complex but there is a change in conformation of RNA [47]. At low temperatures, an increase in the helicity of Rev peptide is observed when it binds selectively to IIB RNA, which again suggest that the binding is coupled to the folding [48]. Tan *et al*. showed that the peptide consisting of residues from this region (corresponding to amino acids 34–50 of HIV Rev protein) is not only responsible for target specificity, but also retains binding affinity as tight as that of the isolated intact protein. Additionally, the peptide is able to bind the Rev responsive element specifically and is sufficient for a high binding affinity, comparable to that of Rev [48–50].

The examples described above represent scenarios where researchers have used intrinsically disordered proteins as models for the design of their peptides. The motive behind the design of these peptides is to mimic the original protein, but engineering synthetic peptides that will mimic the dynamics observed in nature requires careful consideration of the dominant inter- and intra-molecular interactions. Defining the thermodynamics of secondary structure stability will help in understanding the structure-function relationship, leading to better understanding of the overall dynamics observed in nature. The following section will examine simple “rules” that one could apply to design an amphiphilic α-helix.

## 3. Designing Amphiphilic α-Helix Peptide

In order to better understand the fundamental thermodynamics that describes the cooperative phenomenon, a variety of strategies have been successfully employed to define a dynamically folding helix whereupon one can build rapid binding selectivity [51–58]. One simple strategy is to design an amphiphilic α-helix peptide based on a two-part algorithm, where intrinsic propensity and periodicity define helical stability. In 1974, Chou and Fasman [59] defined intrinsic propensity by calculating the conformational parameters of the 20 amino acids from the frequency of occurrence of these different amino acids in α-helix, β-sheets and random coil in 15 proteins (Table 1). This conformational parameter refers to the intrinsic inclination of individual amino acids to a given secondary structure, where side-group van der Waals and steric interactions tend to restrict an amino acid to particular Φ and Ψ conformations.

**Figure 2.**
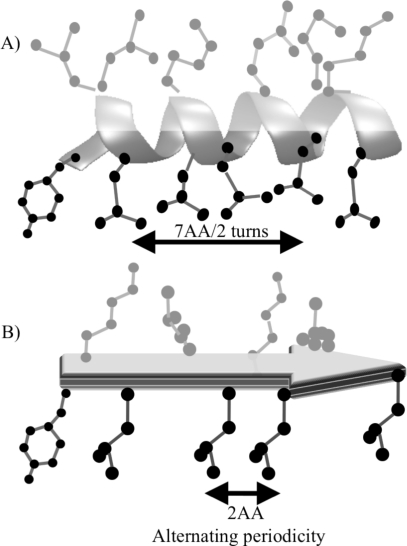
Peptide in α-helix (**A**) and β-sheet (**B**) conformation with polar side chains showed in gray color while non-polar in black color, AA: Amino acid. Amino acid sequence matches the periodicity requirement for α-helix and β-sheet making it amphiphilic (adapted from Xiong *et al*. [61]).

DeGrado and Lear defined periodicity by investigating the role of hydrophobicity in the peptide sequence in determining the secondary structure in bulk and at apolar/water interfaces [60]. These nonlocal interactions are “programmed” into the sequence with a recurring pattern that defines a particular secondary structure. α-Helices have a periodicity of 7 (3.5 amino acid per turn), meaning that the amino acids at *i* and *i* + 7 define the refrain, and β-sheets have two residues per turn (Figure 2). DeGrado and Lear study was based on the design of three synthetic peptides comprising of leucine (L) and lysine (K) with different hydrophobic periodicities and chain lengths. As all the three peptides consist of only two amino acids, leucine and lysine, intrinsic propensity for each peptide is the same and the only difference is the hydrophobic periodicity, and the effect of hydrophobic periodicity on the secondary structure can be investigated (Table 2).

Chou-Fasman parameters for all the three peptides are Pα = 1.19 and Pβ = 1.06, yielding a stronger intrinsic inclination towards α-helix. The hydrophobic periodicity for peptides A and B matches to that of an α-helix, and the periodicity for peptide C matches to that of a β sheet. Circular dichroism was used to measure the secondary structure in bulk, and Langmuir Blodgett technique was used to study the properties of peptides at an air-water interface. Peptide conformation at the air-water interface was determined by compressing the peptide monolayers and transferring them to a solid substrate by using Langmuir Blodgett, and the secondary structure was detected by using infrared and circular dichroism spectroscopy. Circular dichroism in bulk solution showed that the peptide A was too short to form α-helices, peptide B showed α-helix secondary structure, and peptide C formed β-sheets. In Peptide C, the presence of the alternating hydrophobic periodicity appears to override the short-range interactions, and the peptide forms β-sheets, despite the helical propensity. This shows the periodicity of the polar/non-polar residues dominates over the intrinsic propensity.

**Figure 3.**
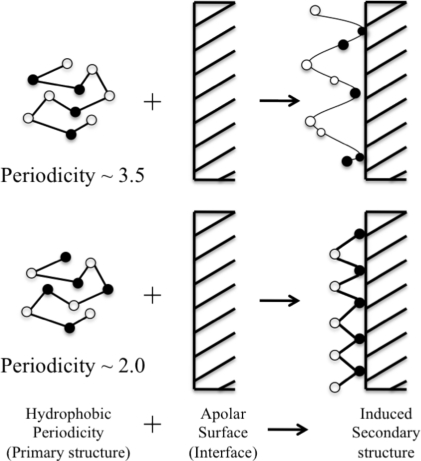
Schematic diagram showing the effect of hydrophobic periodicity on the secondary structure at interfaces. Filled circles are hydrophobic residues and blank circles are hydrophilic residues. Peptides at apolar/water interface arrange in such a way that will maximize the contact between hydrophobic residues and apolar surface and the contact between hydrophilic surface and aqueous environment (adapted from DeGrado *et al*. [60]).

Langmuir Blodgett experiments showed that peptides B and C formed more stable monolayers as compared with peptide A. Due to hydrophobic periodicity, peptides B and C form a more stable secondary structure such that the hydrophobic residues are segregated on one side, forming an apolar surface (Figure 3). At air-water interface, the apolar surface of the peptide will partition into air phase, where the free energy of dehydration of the hydrophobic side chains is the driving force for separation, stabilizing the amphiphilic secondary structure of the peptide.

**Figure 4.**
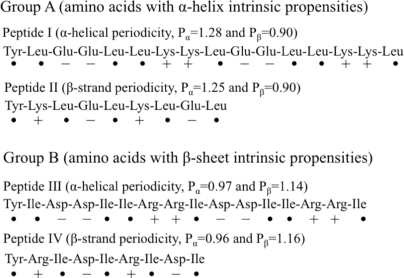
Group A is composed of amino acids with α-helix intrinsic propensities and Group B is composed of amino acids with β-sheet intrinsic propensities. + and −: Polar amino acids; •: non-polar amino acids (Xiong *et al*. [61]).

Xiong *et al*. [61] observed similar results, showing that the periodicity of polar and non-polar residues determines the secondary structure of the given amino acid sequence by comparing the intrinsic propensities of amino acids to polar/non polar periodicity in direct competition. Two groups of peptides (Figure 4) were designed, where group A consisted of Leu, Lys and Glu, and group B consisting of Ile, Arg and Asp. Each group has one hydrophobic residue, one negatively charged residue and one positively charged residue. Amino acids in group A has a intrinsic inclination towards α-helix secondary structure while those in group B have tendency to form β-sheets. In group A, peptide I is “programmed” such that it favors the α-helix secondary structure while periodicity of peptide II opposes the α-helical secondary structure. Similarly, in group B, peptide III is “programmed” such that it favors the α-helix secondary structure while periodicity of peptide IV favors the β-sheet secondary structure. Thus, there are two sets of peptides where the permutations intrinsic inclination versus periodicity can be explored.

Using circular dichroism, Xiong et al shows peptide I has intrinsic inclination (Pα = 1.28, Pβ = 0.90) towards α-helix forms an α-helix, while peptide III has intrinsic inclination towards β-sheet (Pα = 0.97, Pβ = 1.14) but forms an α-helix despite the propensity of the group. These Pα and Pβ values were calculated using the method described in Chou and Fasman [59]. Similarly, peptide II has intrinsic inclination towards an α-helix but forms a β-sheet. Their work corroborates the idea that periodicity of polar and non-polar residues dominates the secondary structure of the sequence.

Stability of the secondary structure is also an important aspect in designing synthetic peptides. The Hodges group investigated the effect of hydrophobicity of amino acids side chains and intrinsic propensities on the stability of an amphiphilic α-helix [62]. It was found that even though hydrophobicity and intrinsic propensities are not correlated with each other, they do contribute to the stability of amphiphilic α-helix. These experiments show that the synergistic effect of intrinsic propensities and hydrophobicity drives the formation of stable helices. DeGrado and O’Neil determined the thermodynamic stabilities of the naturally occurring amino acids in α-helix as against random coil [63] while Lyu *et al*. determined the role of amino acids side chains in stabilizing/destabilizing α-helix [64]. Another important aspect in designing a stable α-helix peptide is the chain length of the sequence. DeGrado and Lear [60] found that helix formation requires 14 residues while β-sheet requires 7 residues. This was proven by the LK peptides described above. Peptide A does not form helices as the periodic sequence becomes shorter, but peptide B forms stable α-helices with two septet repeats. Narita *et al*. observed the similar results for the critical chain requirement for α-helix and β-sheet [65]. Under forcing conditions, low dielectric solvents that favors the secondary structure formation, critical chain length for α-helix is approximately 13, and the critical chain length is four residues for β-sheets.

These studies together show that the primary structure of a peptide can be designed by using intrinsic propensity and periodicity of polar/non-polar amino acids, where upon folding the hydrophobic and hydrophilic domains become spatially disjoint, leading to amphiphilic behavior. Still, the activity of these amphiphilic sequences captures only one aspect discussed in the introduction, namely, conformational selection and not fly-casting. The following section discusses the potential to apply these dynamics to peptide design for selective binding, where one can create novel peptide sequences that have dynamic amphiphilicity by folding in response to environmental cues. Such a peptide with tunable amphiphilicity can be designed to bind to a wide range of targets, coupling dynamic amphiphilicity to selective binding. Moreover, these peptides can take advantage of the accelerated kinetics inherent in natively unfolded structures and applied as a template for next generation biologically inspired molecular designs.

## 4. Engineered Synthetic Peptides

### 4.1. Membrane Mimics

**Figure 5.**
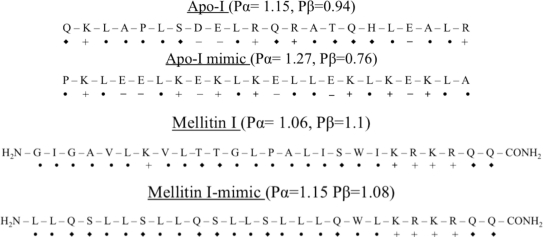
(**A**) Amino acid sequence of Apo I and its analogous synthetic amphiphilic peptide; (**B**) Amino acid sequence of Melittin I and its analogous synthetic amphiphilic peptide. + and −: Polar amino acids; ♦: polar neutral amino acids; •: non-polar amino acids.

Amphiphilicity is an important characteristic of many membrane bound peptides and proteins such as apolipoproteins, peptide hormones, and signal peptides. Plasma Apolipoprotein (Apo-I) consists of six highly homologous 22 amino acid segments, each containing amino acids with a high α-helix propensity and periodicity [66] (Figure 5A). A model docosapeptide is designed by equally distributing acidic and basic residues on the hydrophilic side and amino acids were selected such that the model peptide is different from the repeating Apo-I peptide sequence. The Apo-I mimic peptide has the potential to form an amphiphilic α-helix and has the similar binding characteristic of apolipoprotein A–I by comparing the different criteria such as binding to phospholipid single bilayer vesicles, surface activity and ability to activate lecithin:cholesterol acyltransferase [67,68].

Melittin, a toxic component from bee venom, is a hexacosapeptide in which the *N*-terminal region is predominantly hydrophobic (residues 1–20) and *C*-terminal is predominantly hydrophilic (residues 21–26) (Figure 5B). It has many properties similar to the apolipoproteins as it also binds to phospholipids bilayer, forms stable monolayer at the air-water interface and forms α-helix upon tetramerization or when bound to sodium dodecyl sulphate micelles or phospholipids bilayers [69]. The membrane interface has a potent ability to induce the secondary structure in melittin, and this folding of the peptide is coupled with its partitioning in the membranes. It has been shown that the amphiphilic structure is important for the hemolytic activity of mellitin [70]. DeGrado *et al*. designed the non-homologous analogous amino acid sequence, with hydrophobic:hydrophilic ratio of 2:1, which provides valuable information about the role of hydrophobic-hydrophilic balance in the interaction of amphiphilic peptides with mono- and bilayers. Leucine is selected in the melittin mimic-I sequence because of its hydrophobicity and high α-helix forming tendency, and the leucine is placed on the hydrophobic face of the α-helix while glutamine is selected for the hydrophilic face. Serine residues are included to increase the hydrophobicity of the model peptide such that the amphiphilicity is equivalent to native peptide. This synthetic peptide has higher potential to form amphiphilic α-helix, and the authors found that both peptides, Melittin I and Melittin I-mimic, binds to the unilamellar egg lecithin vesicles and both are capable of disrupting phospholipids bilayer [71,72].

These synthetic analogous amphiphilic helical peptides of simple sequence are a powerful tool for the systematic study of the structure-function relationship of lipid-associated proteins and the construction of water-soluble lipid-peptide complexes of desirable physical and physiological properties. These results also suggest that the rational design of peptides based on secondary structure considerations is a useful tool to elucidate the structure-function relationship in biologically active peptides.

### 4.2. *de Novo* Designs

**Figure 6.**
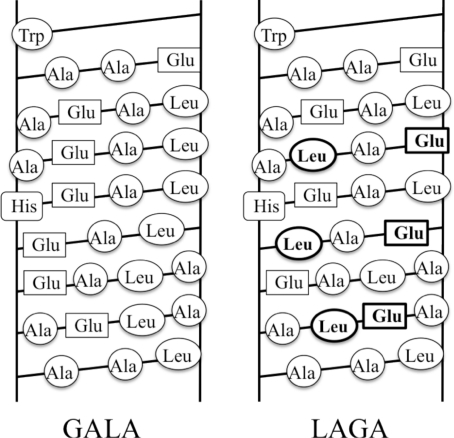
Helical grid representation of the GALA and LAGA peptides designed by Szoka’s group. Difference between these two peptides is the aligning of the glutamic acid and leucines. They are on the different faces of the helix making GALA amphipathic (adapted from Parente *et al*. [63]).

Engineering synthetic peptides that captures both the dynamics of folding without disrupting the selectivity of binding requires careful design considerations. To this end, the Szoka’s group has designed the GALA peptide [73], a 30 amino acid synthetic peptide comprising of glutamic acid-leucine-alanine repeat that mimics the viral activity of hemagglutinin. This peptide switches between a random coil and an amphiphilic α-helix when the pH of the solution is changed from 7 to 5. Glutamic acid is selected as a titratable residue that destabilizes the helix while at low pH and promotes the helix formation at high pH. The hydrophobic face has enough hydrophobicity to interact with the neutral bilayer membranes at low pH [74,75]. There are several papers that investigate the mechanism of pore formation, rates of membrane permeabilization and the role of sequence [76–81]. LAGA peptide is designed from the same amino acids where the only difference is in the positioning of the leucines and glutamic acids (figure 6). LAGA peptide also shows the transition from random coil to α-helix with decreases in pH from 7.5 to 5, but the LAGA peptide does not form an amphiphilic α-helix. When the two peptides were compared, GALA partitions into membrane more effectively than LAGA. Therefore, the GALA peptide can initiate leakage of vesicle contents and membrane fusion [82].

Since GALA peptide can destabilize the bilayer at low pH, it has been used as a carrier for gene delivery. GALA peptide as such cannot bind directly to DNA as both have net negative charge, but when GALA is used with other synthetic amphipathic components, it can be used for DNA or ODN (oligonucleotide) delivery. Various components like polylysine [83], dendrimer [84] and cationic liposomes [85–87] have been used with GALA peptide and transfection efficiency of the DNA delivery has been studied. Design of KALA peptide, a synthetic cationic peptide, is based on GALA where the negatively charged glutamic acid is replaced with positive charge lysine in the similar position in the peptide backbone while maintaining the other properties [88]. The KALA peptide has been designed to deliver DNA without using other amphipathic components. In contrast to the GALA peptide, the KALA peptides goes from α-helix to random coil when the surrounding pH is changed from 7.5 to 5. The reason for this behavior is the net increase in positive charge of the peptide that destabilizes the α-helix when the pH is decreased. Positive charges on KALA peptide can bind directly to DNA, and the hydrophobic fraction of the peptide can interact with the lipid packing of a membrane [89]. These engineered synthetic peptides are capable of interacting with DNA, and they represent a starting point for generating new synthetic molecules that are capable of binding selectively to DNA.

**Figure 7.**
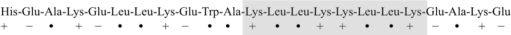
Synthetic peptide designed by Jain *et al*. [90]. Grey colored region shows the positive charge present in the peptide that prevents it from folding in the aqueous solution (Pα = 1.28, Pβ = 0.81). + and −: Polar amino acids; •: non-polar amino acids.

**Figure 8.**
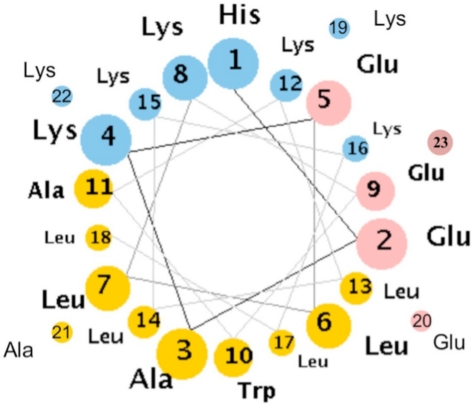
Helical Wheel for the model peptide system, where hydrophobic (yellow), basic (blue) and acidic (red) are highlighted.

Extending the idea of designing binding-folding cooperativity, Jain *et al*. designed a synthetic peptide (Figure 7) template using the rules described above, where dynamics of association can be quantitatively explored [90]. The model peptide design follows from the work in DeGrado’s lab, where they examined the role of hydrophobic periodicity in the leucine-lysine (LK) peptides that defines the secondary structure, in bulk and at air-water interface, and the amphiphilicity. As discussed above, leucine-lysine (LK) peptides shows that the correct periodicity can stabilize amphiphilic secondary structure at hydrophobic interfaces [60]. Jain *et al*. have used amino acids that have inclination to form α-helix secondary structure (Pα = 1.28, Pβ = 0.81) and “programmed” the sequence so that it will form an amphiphilic α-helix (Figure 8). This peptide design is analogous to antibacterial [91] and hemagglutinin peptides [92] found in nature. A 23-chain amino acid is synthesized with 8 positive charges and 5 negative charges.

Circular dichroism (CD) spectroscopy is used to measure the ensemble average secondary structure, showing that the model peptide in deionised water consists of random coil and little α-helix (10%), and, in presence of salt (NaCl), an increase in helicity is observed. The reason for this increase in helicity is due to presence of charge in the peptide, where electrostatic repulsion prevents the peptide from forming an α-helix (Figure 7, grey colored area) in deionised water. In presence of salt (NaCl) or DNA, there is screening of the charges and a decrease in the Debye length.

**Figure 9.**
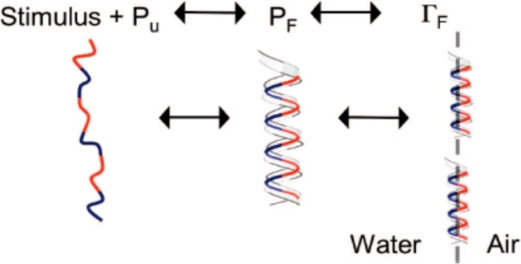
Folding initiated interfacial dynamics. Pu is the population of the unfolded peptide and Pf is for the folded peptide. Blue = hydrophilic, Red = hydrophobic.

The dynamics of this amphiphilic transition is characterized by using pendant bubble apparatus. Pendant bubble is used to measure dynamic surface tension as well as equilibrium surface tension. The technique is often used to measure the dynamics of “regular” surfactant systems. In the case of the model peptide, it is assumed that only folded peptide adsorbs at the air water interface and causes the reduction in surface tension (Figure 9). The effect of different parameters like peptide concentration (with constant salt concentration) and salt concentration (with constant peptide concentration) on the dynamic surface tension were also studied. As the peptide concentration is increased, the rate of change in the surface tension increases. As the salt concentration is increased, the rate of change in surface tension also increases. These results agree with the circular dichroism observations, where the helicity increases with increased salt concentration. Jain *et al*. have numerically modeled the dynamic surface tension (dynamic amphiphilicity) to see the effect of different parameters on the folding of the peptide and the transport of the peptide to the air-water interface [93].

With the model peptide, Jain *et al*. showed that one can design a simple helical peptide template by using the two rules, intrinsic propensity and periodicity, where one can control the stability of the secondary structure of the peptide by manipulating surrounding conditions. This template provides a starting point for engineering systems where these dynamics are coupled to the selective binding to different target sites that can be used for different applications such as bioseparations and drug delivery.

## 5. Conclusions

Natural systems achieve unparalleled degree of selectivity and exceptional rapidity by combining folding behavior with binding in cooperative processes, and an increasing number of scientists are mimicking these natural systems with engineered peptides that exhibit tunable structure and amphiphilicity. This rational design effort requires careful consideration in choosing amino acids and positioning them so that the resulting sequence will have a dynamic secondary structure. Moreover, designing the analogues of peptides observed in nature will elucidate the overall phenomenon, where enhanced kinetics can be incorporated into novel designs. The simple amphiphilic peptide designs presented in this review show the potential for controlling the dynamics of folding of the peptide. We believe that selectivity and kinetics of binding can be built into future designs. Engineering synthetic peptides, where tunable amphiphilicity is coincident with the inherent specificity, will have farreaching benefits for the design of biomimetic tools, particularly in scenarios where fast rates and selective binding in a sea of similar molecules are essential.

## Figures and Tables

**Table 1.** Chou and Fasman’ conformation parameters of the 20 amino acids.

**Helical Residues**	**Pα**	**β-Sheet residues**	**Pβ**
Glu^(−)^	1.53	Met	1.67
Ala	1.45	Val	1.65
Leu	1.34	Ile	1.60
His^(+)^	1.24	Cys	1.30
Met	1.20	Tyr	1.29
Gln	1.17	Phe	1.28
Trp	1.14	Gln	1.23
Val	1.14	Leu	1.22
Phe	1.12	Thr	1.20
Lys^(+)^	1.07	Trp	1.19
Ile	1.00	Ala	0.97
Asp^(−)^	0.98	Arg^(+)^	0.90
Thr	0.82	Gly	0.81
Ser	0.79	Asp^(−)^	0.80
Arg	0.79	Lys^(+)^	0.74
Cys	0.77	Ser	0.72
Asn	0.73	His^(+)^	0.71
Tyr	0.61	Asn	0.65
Pro	0.59	Pro	0.62
Gly	0.53	Glu^(−)^	0.26

**Table 2.** Peptide sequence with hydrophobic periodicities, intrinsic propensity of the sequence and the observed conformation in bulk.

**Peptide**	**Hydrophobic repeat period**	**Intrinsic propensity**	**Result in bulk**
LKKLLKL (A)	3.5	α (Pα = 1.19, Pβ = 1.06)	Sequence too short
(LKKLLKL)_2_ (B)	3.5	α (Pα = 1.19, Pβ = 1.06)	α
LKLKLKL (C)	2.0	α (Pα = 1.19, Pβ = 1.06)	β
